# The temporary and accumulated effects of transcranial direct current stimulation for the treatment of advanced Parkinson’s disease monkeys

**DOI:** 10.1038/srep12178

**Published:** 2015-07-29

**Authors:** Hao Li, Xiaoguang Lei, Ting Yan, Hongwei Li, Baihui Huang, Ling Li, Liqi Xu, Li Liu, Nanhui Chen, Longbao Lü, Yuanye Ma, Lin Xu, Jiali Li, Zhengbo Wang, Baorong Zhang, Xintian Hu

**Affiliations:** 1 Key Laboratory of Animal Models and Human Disease Mechanisms of Chinese Academy of Sciences & Yunnan Province, Kunming Institute of Zoology, Chinese Academy of Sciences, Kunming, Yunnan, 650223, China; 2 CAS Center for Excellence in Brain Science, Chinese Academy of Sciences, Shanghai, 200031, China; 3 Department of Neurology, Second Affiliated Hospital, School of Medicine, Zhejiang University, Hangzhou, Zhejiang, 310009, China; 4 Medical imaging department, Kunming general hospital of PLA, Kunming, Yunnan, 650032, China; 5 Kunming Primate Research Center, Kunming Institute of Zoology, Chinese Academy of Sciences, Kunming, Yunnan, 650223, China; 6 University of Chinese Academy of Sciences, Beijing, 100049, China

## Abstract

Transcranial direct current stimulation (tDCS) is a useful noninvasive technique of cortical brain stimulation for the treatment of neurological disorders. Clinical research has demonstrated tDCS with anodal stimulation of primary motor cortex (M1) in Parkinson’s disease (PD) patients significantly improved their motor function. However, few studies have been focused on the optimization of parameters which contributed significantly to the treatment effects of tDCS and exploration of the underline neuronal mechanisms. Here, we used different stimulation parameters of anodal tDCS on M1 for the treatment of aged advanced PD monkeys induced with 1-methyl-4-phenyl-1, 2, 3, 6-tetrahydropyridine (MPTP) administration, and then analyzed the temporary and accumulated effects of tDCS treatment. The results indicated anodal tDCS on M1 very significantly improved motor ability temporarily; importantly, the treatment effects of anodal tDCS on M1 were quantitatively correlated to the accumulated stimulation instead of the stimuli intensity or duration respectively. In addition, c-fos staining showed tDCS treatment effects activated the neurons both in M1 and substantia nigra (SN). Therefore, we propose that long time and continue anodal tDCS on M1 is a better strategy to improve the motor symptoms of PD than individual manipulation of stimuli intensity or duration.

Parkinson’s disease (PD) is one of the most common neurodegenerative diseases with typical motor symptoms including bradykinesia, postural instability, rigidity and tremor, and characteristic pathological change that dopaminergic neuron death in the substantia nigra pars compacta (SNc)^1^. Over the past few decades, there were major advances on neurological treatments including pharmacological treatments, physical or behavioral therapy, deep brain stimulation (DBS) and noninvasive stimulation approaches^2^. Nonspecific effects, insufficient tailoring to the individual, and moderate to severe adverse effects are significant limitations of pharmacological treatments^2^,^3^,^4^. In physical or behavioral therapy, expertise of the therapist and the patient’s cooperation largely limits its broad implications^2^. Although effective and hopeful to PD treatment^5^, DBS is limited to a small well-defined patient population and carries the risk of serious surgical complications and significant neuropsychiatric side effects^6^,^7^. Noninvasive brain stimulation approaches, such as repetitive transcranial magnetic stimulation (rTMS) and transcranial direct current stimulation (tDCS), are appealing because of their noninvasive, safe and convenience characters^2^. Meanwhile, tDCS carries some advantages over rTMS, including a favorable safety profile, tolerability, easier applicability and cost-effectiveness^6^. Therefore, tDCS could be potentially an ideal choice for PD treatment.

In tDCS, the cerebral cortex is stimulated through a weak direct current in a noninvasive and painless manner^8^. Low-amplitude direct currents penetrate the skull into the brain via scalp electrodes to modify neuronal transmembrane potentials, thereby influencing the levels of excitability and modulating the firing rates of individual neurons^9^,^10^,^11^,^12^, and finally regulate or repair behavioral dysfunctions by modifying activity of excited cortex.^11^,^13^.

Clinical practice has provided little but important information on tDCS treatment for movement symptoms of PD^6^,^8^,^14^. Experimental data also indicated the efficacy of motor cortex stimulation contributed to PD treatment^15^,^16^. Previous studies suggest the most effective stimuli locations are primary motor cortex (M1) and prefrontal cortex (PFC). Exciting M1 may directly affect the motor functions of PD patients, but PFC may be mainly contributed to improve the cognitive functions of PD patients^8^. These studies mentioned above just showed the tDCS treatment for PD patients were significantly effective, but they didn’t explore the different treatment effects according to varies of stimuli parameters^6^,^8^. So far, we still know little about the effective parameters which will significantly regulate the treatment effects of tDCS.

Here, we studied the anodal tDCS treatment effects on M1 with different stimuli parameters to improve motor symptoms in aged advanced PD monkeys (*Macaca mulatta*) induced by MPTP. Non-human primate PD models are widely regarded as essential preclinical investigation for PD treatment^17^,^18^,^19^,^20^,^21^. The common PD model currently available is the classic PD monkey model chronically induced by 1-methyl-4-phenyl-1, 2, 3, 6-tetrahydropyridine (MPTP)^17^,^18^,^22^,^23^. However, one limitation of this model is that monkeys often spontaneously recover from motor symptoms after MPTP intoxication^24^,^25^,^26^. In this study, to avoid the automatic recovery of PD monkeys, we developed an aged (20 years old) advanced PD model with acute intramuscular injections (i.m.) of MPTP-saline solution. The severity of Parkinsonism before and after tDCS treatment was evaluated according to improved Kurlan scale^27^, which was widely accepted as a valued scale for PD research in old world monkeys^28^. C-fos is a nuclear proto-oncogene that has been implicated in many important cellular events^29^,^30^, and this gene is translated to a transcription factor and it is a functional marker of activated neurons^31^,^32^. Recently, c-fos became the most widely used powerful tool to delineate individual neurons as well as extended circuitries that are responsive to wide variety of external stimuli^31^,^33^,^34^,^35^. We used this useful tool to explore the c-fos protein express changes in M1 and SN after tDCS treatment, which might help elucidating mechanisms of tDCS. Finally, we examined the pathological changes of SNc in two MPTP treated monkeys by TH staining to verify the PD model.

## Results

**Figure 1:**
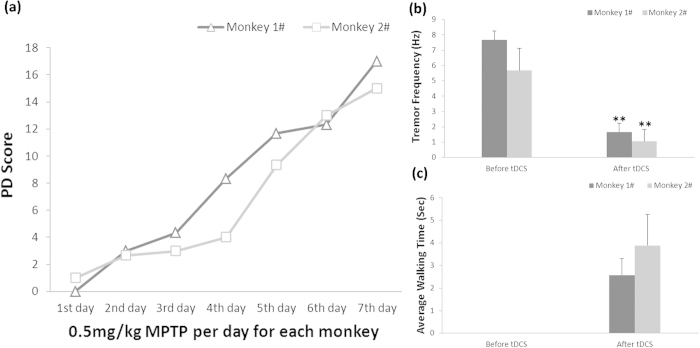
The temporary effects of anodal tDCS on M1 in two advanced PD monkeys (1#, 2#). (**a**) Acute modeling process of MPTP administration. (**b**) Tremor frequency of PD monkeys was dramatically improved by anodal tDCS on M1. (One-way ANOVA, Monkey 1#: **P = 0.0002; Independent Sample Mann-Whitney U Test, Monkey 2#: **P = 3.3 × 10^−9^). (**c**) Voluntary locomotion of PD monkeys was obviously increased after anodal tDCS on M1. Values in (**b**,**c**) represent mean ±STD.

Acute MPTP administration modeling process was showed in Fig. 1(a): two monkeys survived after acute MPTP administration (0.5 mg/kg, 7 daily injections). Tremor frequency and walking time were used to evaluate the treatment effect. Fig. 1(b) showed the tremor frequency of the two advanced PD monkeys temporarily decreased significantly after M1 tDCS (0.3 mA, 10 min). Amazingly, these two advanced PD monkeys could not move before M1 tDCS, but they walked for a while immediately after M1 tDCS (Fig. 1(c)), and they could sit in the cage instead of lying down as before tDCS treatment. These data indicated that M1 tDCS temporarily improved PD monkeys’ motor symptoms dramatically. Unluckily, these two monkeys died after three days, indicating M1 tDCS could not cure PD thoroughly although its temporary treatment effects were excellent.

**Figure 2:**
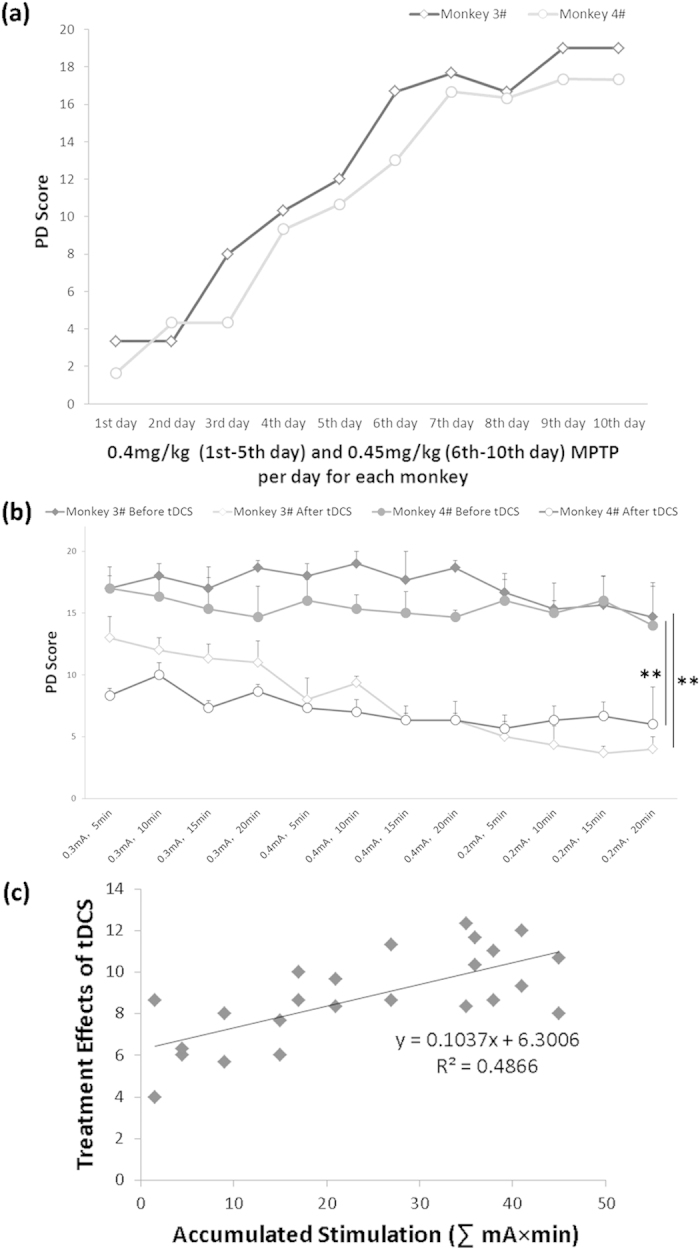
The accumulated effects of anodal tDCS on M1 in two advanced monkeys (3# and 4#). (**a**) Mild-acute modeling process of MPTP administration. (**b**) Consecutive treatment of anodal tDCS on M1. The intensity of tDCS ranges from 0.2 to 0.4 mA and time from 5 to 20 min. (The treatment effect is statistically significant before and after tDCS: Related-Samples Wilcoxon Signed Rank Test for Monkey 3#: **P = 0.002, Paired t-test for Monkey 4#: **P < 0.01). (**c**) The quantitative relationship between accumulated stimulation (∑ intensity × duration) and anodal tDCS treatment effects (PD Score _before tDCS_ − PD Score _after tDCS_) on M1 monkeys 3# and 4#. Grey squares represent the average tDCS treatment effects at different accumulated stimulation of each monkey. Values in (**b**,**c**) represent mean ± STD.

To detect the long-term treatment effects of tDCS and find the main parameters of anodal tDCS on M1 with intensity from 0.3 (referred to the monkeys 1# and 2#), 0.4 (higher) to 0.2 (lower) mA and time from 5, 10, 15 to 20 minutes, another two monkeys (3# and 4#) were used in this experiment (Fig. 2(a)). The PD scores before tDCS showed a mild decrease with stimuli trails, but they were not larger than 2, which meant little medical significance. The PD score of Kurlan scale was dramatically decreased right after anodal tDCS, but was restored to the pretherapeutic level before the next tDCS treatment (Fig. 2(b)). This fast recovery indicated the temporarily therapeutic effects of a single tDCS treatment which disappeared in one day.

However, the changes of PD score after tDCS were significantly decreased over 7 after the consecutive 12-days treatments. Multivariate linear regression was used to evaluate the effects of parameters (monkey, stimuli duration, stimuli intensity, accumulated stimulation) on the decrease. The result showed that the accumulated stimulation (P < 0.01) instead of monkey (P = 0.076), stimuli intensity (P = 0.065) or stimuli duration (P = 0.058) could explain the changes. These results suggested that although single tDCS treatment could not cure PD thoroughly, consecutive treatments of tDCS with accumulated effects were very effective to improve tDCS effect. The more accumulated stimulation of tDCS, the better treatment effect. The relationship between treatment effects (PD Score _before tDCS_ − PD Score _after tDCS_) and accumulated stimulation of tDCS (∑intensity × time) was quantitatively described in Fig. 2(c), which showed a linear increase.

**Figure 3:**
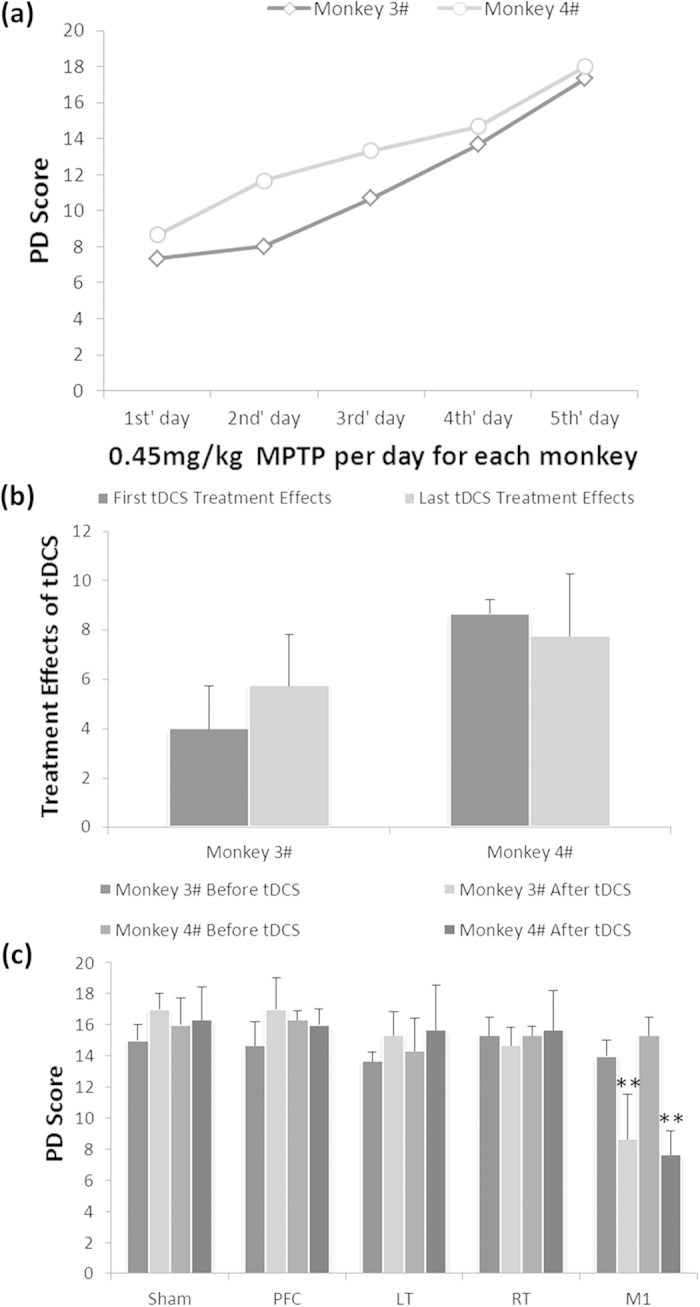
Extinction of tDCS accumulated treatment effects and contributions of different brain areas to tDCS effects for PD treatment. (**a**) After six months’ recovery, monkey 3# and 4# were administered with additional MPTP to restore the Kurlan scale scores same to the scores in Fig. 2(a). (**b**) Extinction of tDCS accumulated treatment effects on M1 after six months’ recovery. There was no significant difference between the effects of the first tDCS and the last tDCS in each monkey (Multi-factor ANOVA: P = 0.881). (**c**) The effects of tDCS treatment (0.2 mA, 5 min) on different brain areas. The PD scores only decreased after tDCS on M1. Sham: control of anodal tDCS on M1 with no stimulation; PFC: prefrontal cortex; LT: left temporal lobe; RT: right temporal lobe; M1: primary motor cortex. Multi-factor ANOVA: The interaction of region × tDCS was significant, **P < 0.01. Values in (**b**,**c**) represent mean ± STD.

Based on the accumulated treatment effects of tDCS, we wanted to know whether the accumulated effect would last at least for six months. After six months’ natural recovery, the PD scores of two monkeys were declined to 5 and 9 respectively. Therefore, the two monkeys received additional MPTP administration (0.45 mg/kg, 5 daily injections) to make sure their PD scores were increased to 16–18 again (Fig. 3(a)). Then the two monkeys were subjected to a single tDCS treatment (0.2 mA, 5 min). The result showed that the therapeutic effect of the single tDCS treatment was not different from the effect of six months before (first tDCS treatment in Fig. 2(b)) (Fig. 3(b)), indicating an extinction of accumulated effect of tDCS six months after. Furthermore, the recovery of PD score in each monkey may be attributed to the compensatory mechanism^24^,^25^,^26^ but not the accumulated effects of anodal tDCS on M1, since the accumulated effect of tDCS treatments was disappeared during the six-month period.

In addition to M1, we also tested other brain areas to ascertain whether these areas contribute to tDCS treatment effects. The results showed that there were no significant difference in treatment effects before and after tDCS treatment on prefrontal cortex (PFC), left temporal lobe (LT) or right temporal lobe (RT) (Fig. 3(c)). These data indicated that stimulation on M1 rather than other brain areas could induce a significant treatment effect of PD motor symptoms.

**Figure 4:**
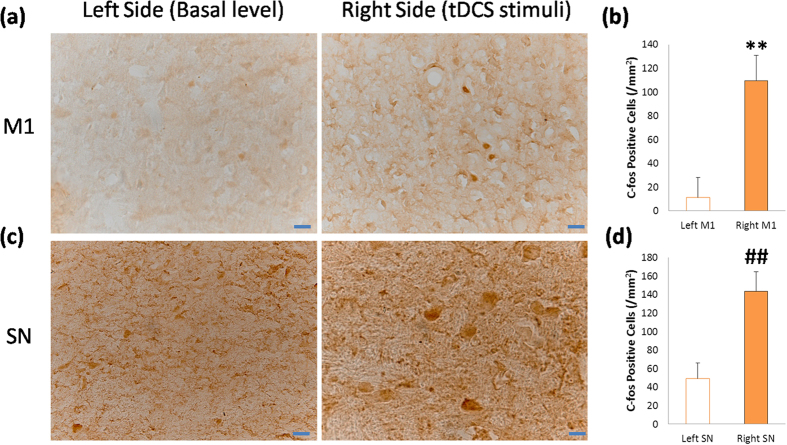
C-fos staining of the neurons in M1 and SN after anodal tDCS on the right side of M1. (**a**) The c-fos immunohistochemical staining of left and right side neurons in M1. C-fos positive cells were counted for analysis. (**b**) The c-fos expression in the neurons of the right side M1 was significantly increased compared to the basal level. Paired t-test: **P = 0.0007. (**c**) The c-fos staining of left and right side neurons in SN. (**d**) The c-fos expression in the neurons of the right side SN was significantly increased. Paired t-test: ##P = 0.0009. Values in (**b**,**d**) represent mean ±STD. Scale bar: 20 um.

Importantly, what is the neuronal mechanism underlying the anodal tDCS on M1? To answer this question, we checked the neuronal activation by c-fos staining after anodal tDCS on M1. Unfortunately, the monkey 3# was died after being finished the experiments above, so we only used monkey 4# for c-fos staining. The most effective and economic design for this study is the self-control in monkey 4#: anodal tDCS on right side of M1, and the left side as the baseline of c-fos expression. Before this experiment, we confirmed the tDCS treatment effect again (the behavior analysis and video clips were provide in Supplementary Data). To avoid unnecessary stimulations activating the c-fos expression, this experiment was done in a silent and dark room. After anodal tDCS on right side of M1 (0.3 mA, 10 min, 3 times repeated), the monkey 4# stayed in the room for 2 hours for the time of c-fos expression, and then was sacrificed for c-fos immunohistochemical examinations. The results showed that tDCS on M1 significantly activated the c-fos expression in the neurons of M1 and SN compared to the basal level (Fig. 4). It is indicated that anodal tDCS on M1 may active the neurons in M1 and SN in the neural circuits associated with motor control, which would improve the PD behaviors.

**Figure 5:**
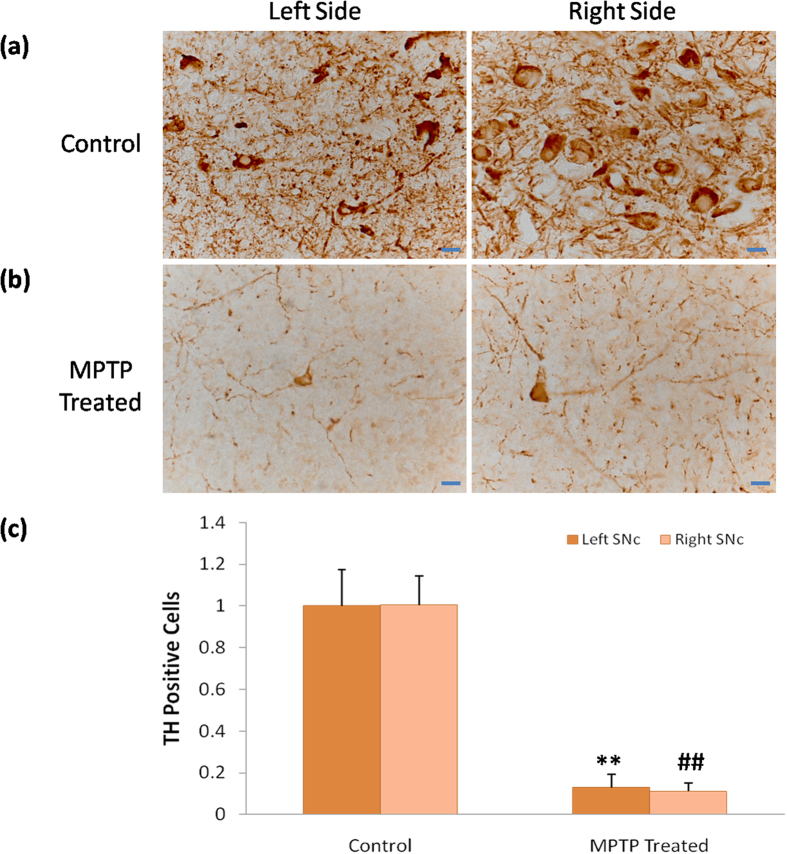
TH staining of bilateral SNc in MPTP treated monkeys and normal control. (**a**,**b**) shows the immunohistochemical images of TH positive cells in the SNc of MPTP treated monkeys and control. (**c**) TH positive cells in the MPTP treated monkeys significantly reduced in the left and right side of SNc compared with control (left: ANOVA, **P < 0.01; right: Independent Sample Mann-Whitney U Test, ##P = 0.001 ), but there was no significant difference between the left and right side of SNc within groups (control: ANOVA, P = 0.961, MPTP treated: ANOVA, P = 0.567). Values in (**c**) represent mean ±STD. Scale bar: 20 um.

Finally, we verified this advanced PD monkey model by TH immunohistochemical staining. The results showed that TH positive neurons in the SNc were significantly lost (more than 80%) in MPTP treated monkeys compared to normal controls, and the morphology of neurons in MPTP treated groups was pathologically changed (Fig. 5). This data was accorded to the advanced PD patients, indicating this model used here was reliable and the tDCS treatment effects were also potentially to be applied in clinic.

## Discussion

To the best of our knowledge, this is the first study to investigate the main factors which modulate the treatment effects of anodal tDCS on M1 for treatment of PD motor symptoms on aged advanced PD monkeys. Firstly, we found that the treatment effect of tDCS was dramatically significant (Fig. 1(b,c)) but not persistent (Fig. 2(b)). We defined this effect as temporary effect of tDCS treatment for PD. Based on the temporary effects of tDCS treatment; we found that the accumulated simulation was a key to facilitate the single temporary effects of M1 tDCS, neither the stimuli intensity nor the stimuli duration (Fig. 2(c)). However, the accumulated effect would become extinct if the consecutive treatment was interrupted for an interval (Fig. 3(b)). The mechanism of extinction of the accumulated tDCS treatment effects was unknown. We speculated that accumulated treatment effect of anodal tDCS on M1 was associated with consecutive electrical stimulation for exciting and facilitating the threshold of M1 excitability. Therefore, we suggested that the effective strategy to use anodal tDCS on M1 as PD treatment was consecutive stimulation with accessible stimuli intensity and time.

This study supports findings efficacy of anodal tDCS on M1, but not on the PFC^8^,^14^. In PD patients, the improvement of PFC functions may repair cognitive processing which would help to finish behavioral tasks better or get higher UPDRS (Unified Parkinson’s Disease Rating Scale) scores. But, the anodal tDCS on PFC of aged advanced PD monkeys in this study showed no significant improvement of motor performances (Fig. 3(c)), the reason was that, although the aged advanced PD monkeys induced with MPTP might be most close to PD patients^36^, the effects of cognitive functions improvement might be exist which could not be detected by using the Kurlan scale.

In anodal tDCS on M1, the M1 was excited by the weak current penetrated in the brain^9^,^10^,^11^,^12^, and then the motor performance was improved. The mechanism that underlies this processing may contribute to the two neural pathways, the direct pathway and the indirect pathway, both from stratum to M1^37^,^38^. Meanwhile, the low current of anodal tDCS on M1 may facilitate M1 activities by means of the activation of N-methyl D-aspartate (NMDA) receptors^39^,^40^. Besides, the role of dopamine involved in anodal tDCS treatment effects on M1 might be accorded to the study that anodal tDCS of M1 prolongs the cortical silent period (CSP)^41^ shown to reflect dopaminergic action in PD^42^,^43^. Although the underlying mechanisms mentioned above has been scientifically demonstrated, the direct neuronal mechanisms are few explored. C-fos is a useful and reliable marker to label the excited neurons in the brain^33^, so it will provide direct neuronal mechanisms of tDCS treatment effects. Our results indicated that anodal tDCS on M1 for the treatment of PD at least activated neurons in M1 and SN (Fig. 4). Interestingly, the c-fos positive areas in neurons of M1 seem to be smaller but darker than that of SN (Fig. 4). As we know, the c-fos mRNA is translated to Fos protein being phosphorylated in cytoplasm and then transferred to the nuclear combined with Jun protein as a transcriptional factor to regulate gene expression^31^,^32^,^44^, so we could conclude that the c-fos staining in M1 neurons was located and aggregated in the nuclear, but the c-fos staining in SN neurons was still distributed in the cytoplasm, indicating the activation of M1 neurons induced by tDCS might be earlier than that of SN. Besides, some research mentioned that the substantia nigra pars compacta receives glutamatergic projections from the medial prefrontal cortex^45^, so tDCS activated M1 may also excite SN neurons. Therefore, M1 was the most effective and broadly used target in tDCS treatment of PD so far. Besides, the TH staining results verified the advanced PD monkey model used here (Fig. 5), confirming the tDCS treatment effects for PD.

In summary, this study provided an indication that long-term and consecutive treatment of anodal tDCS on M1 was more effective than finding the proper stimuli parameters of tDCS for the treatment of PD patients. It would be used in clinical practice and research because of its significant treatment affect in non-human primates PD model.

## Methods

### Monkeys and modeling

The animal cares and experimental protocols were approved by the Ethics Committee of Kunming Institute of Zoology and the Kunming Primate Research Center, Chinese Academy of Sciences (AAALAC accredited), and the methods were carried out in accordance with the approved guidelines^46^,^47^. Eight aged (20 years old, all male) monkeys (*Macaca mulatta*) were selected for this experiment from Kunming Primate Research Center, Kunming Institute of Zoology, Chinese Academy of Sciences. Two monkeys were acutely administrated with MPTP (i.m., 0.5 mg/kg per day for each monkey in consecutive seven days) (Fig. 1). Other two monkeys were mild-acutely administrated with MPTP (i.m., 0.4–0.45 mg/kg per day for each monkey in consecutive ten or five days), showed in Figs 2 and 3. Other four monkeys were died during the experiments because of poor general conditions^48^,^49^. Other two normal monkeys didn’t participate in this study but were sacrificed for TH immunohistochemical staining as control.

### TDCS process

Monkeys were trained to sit in the monkey chairs during the tDCS processes. The location of brain areas such as M1 of each monkey was verified according to the MRI results (MRI was performed on a 1.5 Tesla system, Siemens, Germany) and marked on the surface of scalp. The devices of tDCS were designed by our lab, and the stimuli intensity or time was adjustable (stimuli intensity was ranged from 0–2.5 mA and stimuli time was ranged from 0–60 min). The anodal and cathode electrodes are saline-soaked square rubber pad with sponge insert (4 cm^2^). The location for cathode electrode was always on the occipital lobe, but the location for anodal electrode changed according to the experiments (M1, PFC, LT and RT). Before each tDCS treatment, monkeys were barbered and the scalp was cleaned with saline. Conductive paste was applied to the surface of each electrode, and then the electrodes were fixed on the scalp locations with adhesive tapes. The stimuli intensity and time on monkeys was referenced on clinical research and adjusted by the response of monkeys in the preliminary experiments.

### Behavior data analysis

Video recordings of monkeys’ behavior were collected before and after each tDCS treatment for at least 40 minutes with digital cameras (Sony HDR-XR260, Japan) and evaluated for Parkinsonian symptoms using part A of the Kurlan scale, which utilizes seven important measures of PD. Because of the temporary effects of tDCS, data was analyzed with the three consecutive 10 minutes in average. The video collection and analysis were double-blinded to each experimenter.

### C-fos immunohistochemical staining

Monkey 4# was restrained in the monkey chair and treated with anodal tDCS on the right side of M1 (0.3 mA, 10 min, 3 times repeated) in a silence and dark room to avoid unnecessary stimulations in the environment. After that, the monkey was stayed in the room for 2 hours, and then euthanized (1 mL ketamine, 0.15 g/mL, i.m.) for perfusion with 500 mL saline and 500 mL 4% polyformaldehyde-0.01 M PBS. The brain was removed and fixed in 4% polyformaldehyde-0.01 M PBS for one week, and then gradually equilibrated with 20% and 30% sucrose. Coronal sections of the M1 and SN were obtained by sectioning the tissue on a freezing microtome (Leica, CM1850UV-1-1). Slice thickness was set at 20 μm.

Slices were incubated in 0.2% triton X-100 (Solarbio, Beijing, China) 0.01 M PBS for 30 minutes, and were blocked with 2% BSA for 40 minutes at room temperature and then incubated with 1° antibody (1:800 anti-c-fos polyclonal rabbit antibody 226003, synaptic system , Germany) at 4 °C overnight. Slices were rinsed with 0.01 M PBS and then incubated in polymer helper (ZSGB-BIO PV-9000, Beijing, China) for 20 minutes at 37 °C, and then incubated in polyperoxidase-anti-mouse/rabbit IgG (ZSGB-BIO PV-9000, Beijing, China) for 30 minutes at 37 °C. DAB (DAB-1031 Kit, Maixin, Fuzhou, China) was used for coloration of the slices. Slices were dehydrated with an alcohol gradient and made transparent with TO. Neutral balsam was used to close the slides and digital pictures were collected on a microscope (microscope: Olympus, CX41; camera: Olympus DP25; software: CellSens Entry 1.4.1; Japan).

### TH immunohistochemical staining

Slices were incubated in 3% triton X-100 (Solarbio, Beijing, China) 0.01 M PBS for 4 minutes, and were blocked with 10% sheep serum for 15 minutes at room temperature and then incubated with 1° antibody (1:1000 Anti -Tyrosine Hydroxylase AB152, Millipore, USA) at 4 °C overnight. Slices were rinsed with 0.01 M PBS (Solarbio, Beijing, China) and then incubated in biotin conjugated 2° antibody (1:150, Maixin, Fuzhou, China) for 30 minutes, and then incubated in streptavidin conjugated HRP (1:150, Maixin, Fuzhou, China) for 15 minutes. DAB (DAB-1031 Kit, Maixin, Fuzhou, China) was used for coloration of the slices. Slices were dehydrated with an alcohol gradient and made transparent with TO. Neutral balsam was used to close the slides and digital pictures were collected on a microscope, as described above.

### Statistics

One-way ANOVA or Independent Sample Mann-Whitney U Test (non-homogeneous variance) was used to compare the means/distributions of tremor frequency before and after tDCS. Related-Samples Wilcoxon Signed Rank Test (non-homogeneous variance) or Paired t-test (homogeneous variance) was used to compare the means of PD scores before and after tDCS. Multivariate linear regression model was used to extract the main parameters which may impact the effect of tDCS. Multi-factor ANOVA was used to compare the tDCS treatment effects between six months before and after, and to compare the PD scores between different cerebral regions and between before and after tDCS. Immunohistochemical images analysis: in TH staining statistics, 4 slices of each monkey’s SN area were collected and checked by ANOVA (homogeneous variance), and Independent Sample Mann-Whitney U Test (non-homogeneous variance); in c-fos staining analysis, 5 slices were selected from both left and right side of M1 and SN in monkey 4#, and they were checked by Paired t-test (homogeneous variance).

## Additional Information

**How to cite this article**: Li, H. *et al.* The temporary and accumulated effects of transcranial direct current stimulation for the treatment of advanced Parkinson's disease monkeys. *Sci. Rep.* **5**, 12178; doi: 10.1038/srep12178 (2015).

## Supplementary Material

Supplementary Data

Supplementary Movie

## References

[b1] Lees A. J., Hardy J. & Revesz T. Parkinson’s disease. Lancet 373, 2055–66 (2009). 19524782 10.1016/S0140-6736(09)60492-X

[b2] Fregni F. & Pascual-Leone A. Technology insight: noninvasive brain stimulation in neurology-perspectives on the therapeutic potential of rTMS and tDCS. Nat Clin Pract Neurol 3, 383–93 (2007). 17611487 10.1038/ncpneuro0530

[b3] Rascol O. *et al.* A Five-Year Study of the Incidence of Dyskinesia in Patients with Early Parkinson’s Disease Who Were Treated with Ropinirole or Levodopa. New England Journal of Medicine 342, 1484–1491 (2000). 10816186 10.1056/NEJM200005183422004

[b4] Bezard E., Brotchie J. M. & Gross C. E. Pathophysiology of levodopa-induced dyskinesia: Potential for new therapies. Nat Rev Neurosci 2, 577–588 (2001). 11484001 10.1038/35086062

[b5] Perlmutter J. S. & Mink J. W. Deep brain stimulation. Annu Rev Neurosci 29, 229–57 (2006). 16776585 10.1146/annurev.neuro.29.051605.112824 PMC4518728

[b6] Benninger D. H. *et al.* Transcranial direct current stimulation for the treatment of Parkinson’s disease. J Neurol Neurosurg Psychiatry 81, 1105–11 (2010). 20870863 10.1136/jnnp.2009.202556 PMC4162743

[b7] Limousin P. & Martinez-Torres I. Deep brain stimulation for Parkinson’s disease. Neurotherapeutics 5, 309–19 (2008). 18394572 10.1016/j.nurt.2008.01.006 PMC5084172

[b8] Fregni F. *et al.* Noninvasive cortical stimulation with transcranial direct current stimulation in Parkinson’s disease. Mov Disord 21, 1693–702 (2006). 16817194 10.1002/mds.21012

[b9] Nitsche M. A. & Paulus W. Excitability changes induced in the human motor cortex by weak transcranial direct current stimulation. J Physiol 527 Pt 3, 633–9 (2000). 10990547 10.1111/j.1469-7793.2000.t01-1-00633.x PMC2270099

[b10] Priori A., Berardelli A., Rona S., Accornero N. & Manfredi M. Polarization of the human motor cortex through the scalp. Neuroreport 9, 2257–60 (1998). 9694210 10.1097/00001756-199807130-00020

[b11] Wagner T. *et al.* Transcranial direct current stimulation: a computer-based human model study. Neuroimage 35, 1113–24 (2007). 17337213 10.1016/j.neuroimage.2007.01.027

[b12] Miranda P. C., Lomarev M. & Hallett M. Modeling the current distribution during transcranial direct current stimulation. Clin Neurophysiol 117, 1623–9 (2006). 16762592 10.1016/j.clinph.2006.04.009

[b13] Fregni F., Simon D. K., Wu A. & Pascual-Leone A. Non-invasive brain stimulation for Parkinson’s disease: a systematic review and meta-analysis of the literature. J Neurol Neurosurg Psychiatry 76, 1614–23 (2005). 16291882 10.1136/jnnp.2005.069849 PMC1739437

[b14] Doruk D., Gray Z., Bravo G. L., Pascual-Leone A. & Fregni F. Effects of tDCS on executive function in Parkinson’s disease. Neurosci Lett 582, 27–31 (2014). 25179996 10.1016/j.neulet.2014.08.043

[b15] Canavero S. *et al.* Extradural motor cortex stimulation for advanced Parkinson disease. Report of two cases. J Neurosurg 97, 1208–11 (2002). 12450046 10.3171/jns.2002.97.5.1208

[b16] Drouot X. *et al.* Functional recovery in a primate model of Parkinson’s disease following motor cortex stimulation. Neuron 44, 769–78 (2004). 15572109 10.1016/j.neuron.2004.11.023

[b17] Beal M. F. Experimental models of Parkinson’s disease. Nat Rev Neurosci 2, 325–34 (2001). 11331916 10.1038/35072550

[b18] Emborg M. E. Nonhuman primate models of Parkinson’s disease. ILAR J 48, 339–55 (2007). 17712221 10.1093/ilar.48.4.339

[b19] Blandini F. & Armentero M. T. Animal models of Parkinson’s disease. FEBS J 279, 1156–66 (2012). 22251459 10.1111/j.1742-4658.2012.08491.x

[b20] Le W., Sayana P. & Jankovic J. Animal models of Parkinson’s disease: a gateway to therapeutics? Neurotherapeutics 11, 92–110 (2014). 24158912 10.1007/s13311-013-0234-1 PMC3899493

[b21] Blesa J., Phani S., Jackson-Lewis V. & Przedborski S. Classic and new animal models of Parkinson’s disease. J Biomed Biotechnol 2012, 845618 (2012). 22536024 10.1155/2012/845618 PMC3321500

[b22] Duty S. & Jenner P. Animal models of Parkinson’s disease: a source of novel treatments and clues to the cause of the disease. Br J Pharmacol 164, 1357–91 (2011). 21486284 10.1111/j.1476-5381.2011.01426.x PMC3229766

[b23] Kopin I. J. & Markey S. P. MPTP toxicity: implications for research in Parkinson’s disease. Annu Rev Neurosci 11, 81–96 (1988). 3129982 10.1146/annurev.ne.11.030188.000501

[b24] Mounayar S. *et al.* A new model to study compensatory mechanisms in MPTP-treated monkeys exhibiting recovery. Brain 130, 2898–914 (2007). 17855373 10.1093/brain/awm208

[b25] Boulet S. *et al.* Behavioral recovery in MPTP-treated monkeys: neurochemical mechanisms studied by intrastriatal microdialysis. J Neurosci 28, 9575–84 (2008). 18799689 10.1523/JNEUROSCI.3465-08.2008 PMC6671121

[b26] Elsworth J. D. *et al.* Striatal dopaminergic correlates of stable parkinsonism and degree of recovery in old-world primates one year after MPTP treatment. Neuroscience 95, 399–408 (2000). 10658619 10.1016/s0306-4522(99)00437-6

[b27] Smith R. D., Zhang Z., Kurlan R., McDermott M. & Gash D. M. Developing a stable bilateral model of parkinsonism in rhesus monkeys. Neuroscience 52, 7–16 (1993). 8433810 10.1016/0306-4522(93)90176-g

[b28] Imbert C., Bezard E., Guitraud S., Boraud T. & Gross C. E. Comparison of eight clinical rating scales used for the assessment of MPTP-induced parkinsonism in the Macaque monkey. J Neurosci Methods 96, 71–6 (2000). 10704673 10.1016/s0165-0270(99)00184-3

[b29] Holt J. T., Gopal T. V., Moulton A. D. & Nienhuis A. W. Inducible production of c-fos antisense RNA inhibits 3T3 cell proliferation. Proc Natl Acad Sci USA 83, 4794–8 (1986). 3523478 10.1073/pnas.83.13.4794 PMC323828

[b30] Nishikura K. & Murray J. M. Antisense RNA of proto-oncogene c-fos blocks renewed growth of quiescent 3T3 cells. Mol Cell Biol 7, 639–49 (1987). 3547078 10.1128/mcb.7.2.639-649.1987 PMC365119

[b31] Kovacs K. J. c-Fos as a transcription factor: a stressful (re)view from a functional map. Neurochem Int 33, 287–97 (1998). 9840219 10.1016/s0197-0186(98)00023-0

[b32] Herrera D. G. & Robertson H. A. Activation of c-fos in the brain. Prog Neurobiol 50, 83–107 (1996). 8971979 10.1016/s0301-0082(96)00021-4

[b33] Zhang J. *et al.* c-fos regulates neuronal excitability and survival. Nat Genet 30, 416–20 (2002). 11925568 10.1038/ng859

[b34] Sagar S. M., Sharp F. R. & Curran T. Expression of c-fos protein in brain: metabolic mapping at the cellular level. Science 240, 1328–31 (1988). 3131879 10.1126/science.3131879

[b35] Do-Monte F. H., Quinones-Laracuente K. & Quirk G. J. A temporal shift in the circuits mediating retrieval of fear memory. Nature 519, 460–3 (2015). 25600268 10.1038/nature14030 PMC4376623

[b36] Collier T. J., Kanaan N. M. & Kordower J. H. Ageing as a primary risk factor for Parkinson’s disease: evidence from studies of non-human primates. Nat Rev Neurosci 12, 359–66 (2011). 21587290 10.1038/nrn3039 PMC3387674

[b37] Kopin I. J. Parkinson’s disease: past, present, and future. Neuropsychopharmacology 9, 1–12 (1993). 8397719 10.1038/npp.1993.39

[b38] van Eimeren T. & Siebner H. R. An update on functional neuroimaging of parkinsonism and dystonia. Curr Opin Neurol 19, 412–9 (2006). 16914982 10.1097/01.wco.0000236623.68625.54

[b39] Liebetanz D., Nitsche M. A., Tergau F. & Paulus W. Pharmacological approach to the mechanisms of transcranial DC-stimulation-induced after-effects of human motor cortex excitability. Brain 125, 2238–47 (2002). 12244081 10.1093/brain/awf238

[b40] Nitsche M. A. *et al.* Pharmacological modulation of cortical excitability shifts induced by transcranial direct current stimulation in humans. J Physiol 553, 293–301 (2003). 12949224 10.1113/jphysiol.2003.049916 PMC2343495

[b41] Lang N., Nitsche M. A., Paulus W., Rothwell J. C. & Lemon R. N. Effects of transcranial direct current stimulation over the human motor cortex on corticospinal and transcallosal excitability. Exp Brain Res 156, 439–43 (2004). 14745467 10.1007/s00221-003-1800-2

[b42] Wu A. D., Petzinger G. M., Lin C. H., Kung M. & Fisher B. Asymmetric corticomotor excitability correlations in early Parkinson’s disease. Mov Disord 22, 1587–93 (2007). 17523196 10.1002/mds.21565

[b43] Chen R., Garg R. R., Lozano A. M. & Lang A. E. Effects of internal globus pallidus stimulation on motor cortex excitability. Neurology 56, 716–23 (2001). 11274304 10.1212/wnl.56.6.716

[b44] Caputto B. L., Cardozo Gizzi A. M. & Gil G. A. c-Fos: an AP-1 transcription factor with an additional cytoplasmic, non-genomic lipid synthesis activation capacity. Biochim Biophys Acta 1841, 1241–6 (2014). 24886961 10.1016/j.bbalip.2014.05.007

[b45] Blandini F., Nappi G., Tassorelli C. & Martignoni E. Functional changes of the basal ganglia circuitry in Parkinson’s disease. Prog Neurobiol 62, 63–88 (2000). 10821982 10.1016/s0301-0082(99)00067-2

[b46] Tieu K. A guide to neurotoxic animal models of Parkinson’s disease. Cold Spring Harb Perspect Med 1, a009316 (2011). 22229125 10.1101/cshperspect.a009316 PMC3234449

[b47] Kilkenny C., Browne W. J., Cuthill I. C., Emerson M. & Altman D. G. Improving bioscience research reporting: the ARRIVE guidelines for reporting animal research. PLoS Biol 8, e1000412 (2010). 20613859 10.1371/journal.pbio.1000412 PMC2893951

[b48] Gerlach M. & Riederer P. Animal models of Parkinson’s disease: an empirical comparison with the phenomenology of the disease in man. J Neural Transm 103, 987–1041 (1996). 9013391 10.1007/BF01291788

[b49] Potts L. F. *et al.* Modeling Parkinson’s disease in monkeys for translational studies, a critical analysis. Exp Neurol 256, 133–43 (2014). 24070854 10.1016/j.expneurol.2013.09.014 PMC3962841

