# Supplementary material for: The temporary and accumulated effects of transcranial direct current stimulation for the treatment of advanced Parkinson’s disease monkeys
Source: Sci Rep. 2015 Jul 29;5:12178. doi: 10.1038/srep12178 (PMC4518219; doi:10.1038/srep12178)
Supplement: Supplementary Data [file srep12178-s1.doc]

Supplementary data:

**The temporary and accumulated effects of transcranial direct current stimulation for the treatment of advanced Parkinson’s disease** **monkeys**

Hao Li^1,6^,*, Xiaoguang Lei^3^,*, Ting Yan^1^,*, Hongwei Li^1^, Baihui Huang^1,6^, Ling Li^4^, Liqi Xu^4^, Li Liu^4^, Nanhui Chen^1^ , Longbao Lü^5^, Yuanye Ma^1,5^, Lin Xu^1,2^, Jiali Li^1,2^ Baorong Zhang^3^,†, Zhengbo Wang^1^, †, and Xintian Hu^1,2,5^,†

^1^Key Laboratory of Animal Models and Human Disease Mechanisms of Chinese Academy of Sciences & Yunnan Province, Kunming Institute of Zoology, Chinese Academy of Sciences, Kunming, Yunnan, 650223, China.

^2^CAS Center for Excellence in Brain Science, Chinese Academy of Sciences, 320 Yue Yang Road, Shanghai, 200031, China.

^3^Department of Neurology, Second Affiliated Hospital, School of Medicine, Zhejiang University, Hangzhou, Zhejiang, 310009, China.

^4^Medical imaging department, Kunming general hospital of PLA, Kunming, Yunnan, 650032, China

^5^Kunming Primate Research Center, Kunming Institute of Zoology, Chinese Academy of Sciences, Kunming, Yunnan, 650223, China.

^6^University of Chinese Academy of Sciences, Beijing, 100049, China.

* These authors contributed equally to this work.

†Corresponding to: brzhang@zju.edu.cn, wangzhengbo1226@gmail.com or xthu@mail.kiz.ac.cn.


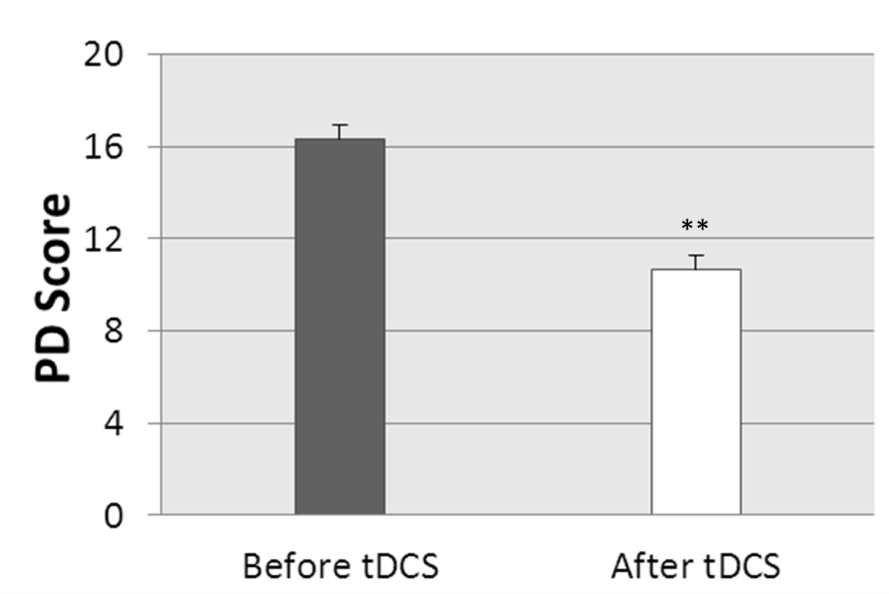


Figure S1. Reconfirmation of tDCS (0.3mA, 10min, 3 times repeated) treatment effects on monkey 4#. The Kurlan score of monkey 4# in cage was significantly decreased after anodal tDCS on M1 (paired t-test, **: P = 0.0034). This data indicated that the monkey 4# was still in the stage of advanced PD without recovery and sensitive to tDCS treatment. This efficacy of tDCS treatment ensured the following study of c-fos staining. The video of this significant treatment effect was also provided in supplementary data.
